# Equivalent circuit models for a biomembrane impedance sensor and analysis of electrochemical impedance spectra based on support vector regression

**DOI:** 10.1007/s11517-019-01970-7

**Published:** 2019-04-02

**Authors:** Ying Xu, Chao Li, Wanxin Mei, Miao Guo, Yong Yang

**Affiliations:** College of Life Information Science and Instrument Engineering, Hangzhou Dianzi University, No. 2 Road, Hangzhou, Zhejiang Province China

**Keywords:** Cell-nitrocellulose membrane, Electrochemical impedance spectra, Equivalent circuit models

## Abstract

In this study, an electrochemical impedance biosensor was developed as a simple and fast method for real-time monitoring of biofilm binding properties via continuous impedance spectroscopy. To prepare the sensing membrane, cells were immobilized onto gold electrodes with nitrocellulose membranes. Different cell growth features were measured with the impedance instrument and analyzed using an equivalent model for data fitting and support vector regression (SVR) for data processing. The collected impedance spectra revealed that the binding attachment areas of cells differ depending on the cell density. Our results demonstrate the usefulness and feasibility of training our impedance-based sensor with a small amount of data to predict the effective area of different biofilms (GE, NGE, and CNGE), with a prediction error of 9.8%.

**Figure:**
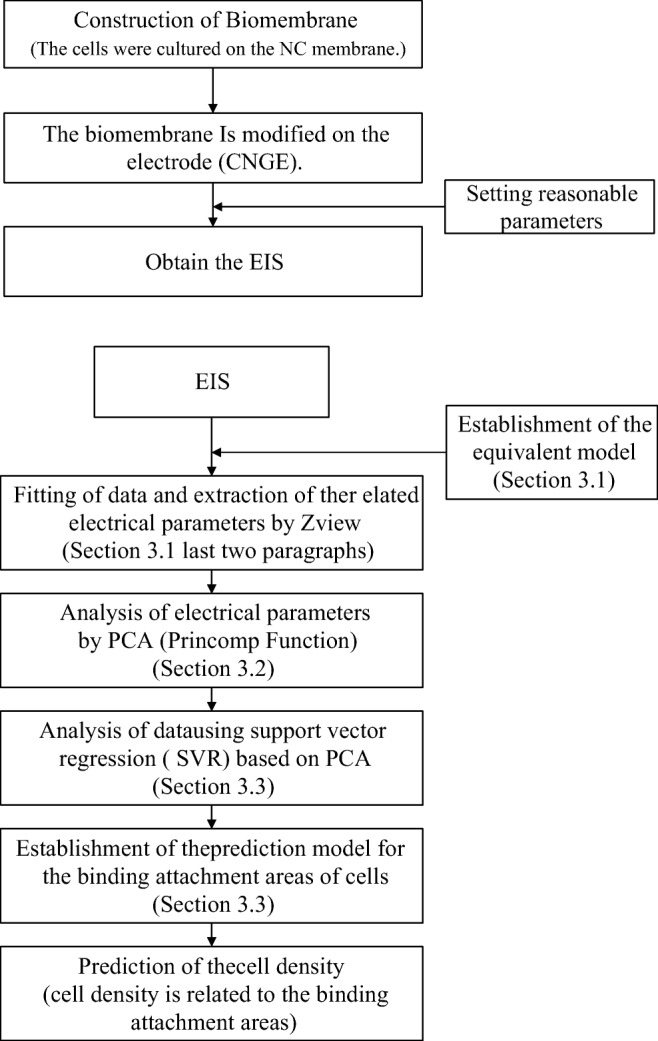
Graphical abstract

Graphical abstract

## Introduction

Electrochemical biosensors, often referred to as potentiometric, amperometric, impedimetric, or conductimetric sensors, are advantageous because they are highly sensitive, rapid, and inexpensive sensors that are also suitable for designing integrated microsystems [1]. The parameters obtained with electrochemical impedance spectroscopy (EIS) can represent different physical quantities [2–5]; thus, EIS can be used as a rapid, label-free, and sensitive process that meets the requirements of microscopic testing [4, 6]. In electrochemical studies, qualitatively and even quantitatively measuring the physical quantities that correspond to the EIS parameters is important [6–8]. A new type of biological sensor was fabricated from an electrode, and an electrochemical biosensor membrane was formed by combining stationary substances with the corresponding detection substances to detect DNA and other biomolecules [9–11]. In addition, the relationship between the electrodes and the biofilm attachment can be used to study the active state of a biomembrane on an electrode surface [10, 12, 13]. Previous studies have shown that the fitted model of this relationship has good stability and prediction performance under different experimental conditions, and the model can predict the effective area of an electrode and provide a method for predicting the active surface area of a biomembrane electrode, which can reflect characteristics of the cell growth status, such as cell density [14–16].

A support vector machine (SVM) is a machine learning algorithm with a wide range of applications; it is especially good at building and forecasting two classification models. Support vector regression (SVR) is a regression method based on a SVM [17, 18]. SVR has been widely used, always predicts data with high accuracy and can be used for quantitative analysis of physical and chemical quantities such as capacitance and resistance [19–21]. However, SVR has not been applied to the analysis of EIS for biosensors. In the present study, SVR is shown to have good accuracy and stability for predicting the active surface area on a biomembrane electrode.

The present work describes the use of EIS for equivalent circuit modeling and a comparison of electrochemical impedance data obtained with three different electrodes: a bare gold electrode (GE), a gold electrode with a nitrocellulose membrane (NGE), and a gold electrode with a cell-nitrocellulose membrane (CNGE). Based on the experimental data, we built an impedance model to obtain the required electrochemical parameters and fit the data with ZView (ZView Version 2.1c, Scribner Associates, Inc.). The main parameter was extracted as the eigenvector of the SVR by evaluating the impedance parameters using principal component analysis (PCA), which is a statistical procedure that uses an orthogonal transformation to convert a set of observations of possibly correlated variables into a set of linearly uncorrelated variables. Finally, the main parameter was used for data training and regression prediction.

## Methods

### Materials

NaCl, KCl, Na_2_HPO_4_·12H_2_O, KH_2_PO_4_, K_3_[Fe (CN)_6_], and K_4_[Fe (CN)_6_]·3H_2_O were purchased from Sinopharm Chemical Reagent Co., Ltd.; nitrocellulose filter membranes were purchased from Biosharp Co., Ltd.; and human breast cancer cells (MCF-7) were purchased from Life Science Institute of Zhejiang University. All chemicals used in this work were analytical grade and used as received. All aqueous solutions were prepared using ultrapure water (ultrapure water produced by Aquelix 5).

### Preparatory experiments

1. Preparation of a 1 mol/L PBS solution: 8 g of NaCl, 0.2 g of KCl, 3.63 g of Na_2_HPO_4_·12H_2_O, and 0.24 g of KH_2_PO_4_ were dissolved in 900 mL of deionized water, and the pH was adjusted to 7.4 using a hydrochloric acid solution. The solution was subsequently transferred to a 1-L volumetric flask and brought to its final volume with deionized water.
2. Preparation of a 50 mM buffer solution: 0.8231 g of K_3_[Fe (CN)_6_] and 0.9200 g of K_4_[Fe (CN)_6_]·3H_2_O were dissolved in a prepared PBS solution and brought to 100 mL.
3. Pre-treatment of electrodes: To ensure that the electrodes were flat and parallel, they were polished and then rinsed with deionized water. Subsequently, the electrodes were examined by cyclic voltammetry. The initial voltage was set to −0.5 V, the final voltage was set to 0.9 V, and the scanning rate was set to 0.05 V/s. For subsequent operations, the difference in the redox peaks had to be less than 0.100 V.
4. Production of the cell-NC membrane: After a clean benchtop was sterilized for 15 min under continuous ultraviolet radiation, one test tube of MCF-7 cells (human breast cancer cell line, Life Science Institute of Zhejiang University) was taken from a liquid nitrogen box. Subsequently, the test tube was thawed in a 37 °C thermostated water bath and then placed on the sterile benchtop. A 1 mL suspension was pipetted from the test tube into a centrifuge tube, and 4 mL of RPMI 1640 medium was added to the tube, which was subsequently placed into a low-speed centrifuge. The sample was centrifuged for 5 min at 800 r/min, and its contents were then carefully mixed. Prior to the cells being transferred into a sterilized cell culture flask, the supernatant was discarded. Then, 4 mL of RPMI 1640 medium was added to the centrifuge tube and mixed. The flask was placed in a constant-temperature CO_2_ incubator, and the cells were cultured for 24 h. Before the cell medium was exchanged, the cells were checked to ensure that they were in good condition. Afterwards, the cells were cultured for another 24 h.

**Fig. 1:**
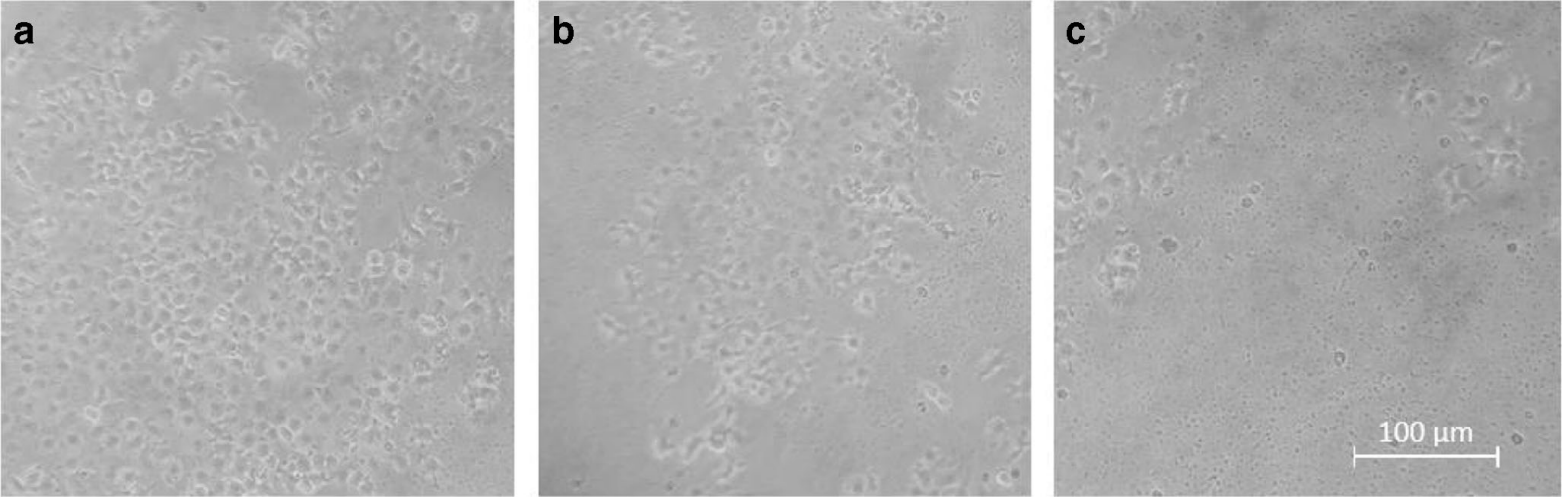
MCF-7 cells of different densities (**a**) high growth density, (**b**) modest growth density, and (**c**) low cell density

The NC membranes (NCEs) were cut into 3 × 3 cm^2^ that were set aside after moist heat sterilization. Well-grown adherent cells were then selected, and the original culture solution was discarded. After digestion, a single-cell suspension was prepared from the original culture solution. Finally, 1 mL of the single-cell suspension was pipetted into a culture dish with the already prepared NC membranes, and RPMI 1640 was added to the culture dish. Afterwards, the culture dish was carefully placed in a CO_2_ incubator at 37 °C for 3 days (Fig. 1). For subsequent electrochemical experiments, an NC membrane with a modest growth density was selected (Fig. 1(b)).

### Electrochemical impedance spectroscopy

The EIS experiment consisted of four main steps—an impedance test, data fitting, PCA and SVR—to gradually construct a biomembrane-electrode system.

Electrochemical measurements were performed using a CH Instruments 760E electrochemical analyzer (CH Instruments Inc., USA). One pole of the electrode was connected to the sensing probe on the electrochemical workstation, and the other pole was connected to both the reference and counter probes on the electrochemical workstation.

**Fig. 2:**
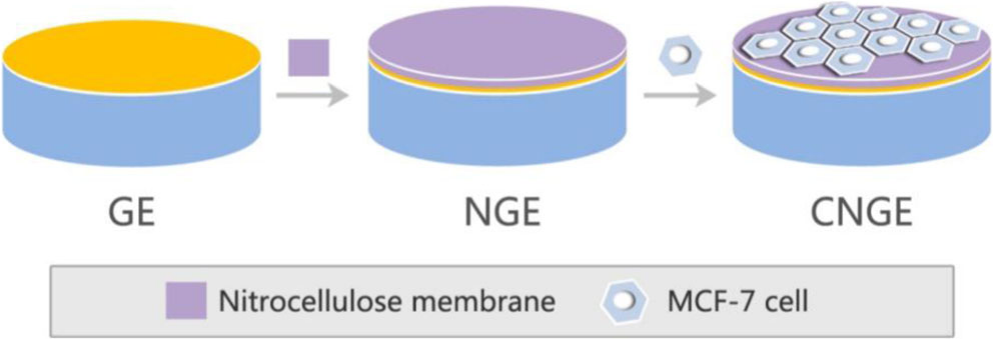
Schematic illustration of GE, NGE, and CNGE

The tested frequency was set in the range from 1 Hz to 100 kHz with a 50 mV AC voltage in the impedance measurement program. Electrodes with cells at different densities were tested in wells containing 50 mM K_4_[Fe (CN)_6_]/K_3_[Fe (CN)_6_] (1:1). For each condition (3 cell conditions × 5 different electrodes), the EIS measurements were recorded 15 times, and the average value was used. After each measurement, the sensor was rinsed with PBS. All electrochemical measurements were performed at room temperature (18–25 °C). To form a three-electrode system for impedance testing, we used a bare gold electrode, an NC film gold electrode, or a cell-NC film gold electrode as the working electrode, a platinum wire electrode as the counter electrode, and a silver/silver chloride electrode as the reference electrode. As shown in Fig. 2, the three experimental groups were recorded as GE, NGE, and CNGE, respectively.

The electrodes in each group were prepared with five different diameters of 1 mm, 2 mm, 3 mm, 4 mm, and 5 mm for the impedance tests. In the NGE electrochemical impedance experiments, the NC film was attached to gold electrodes with different diameters to form an NC-membrane-electrode system for impedance testing. In the CNGE electrochemical experiment, NC membranes with different concentrations of MCF-7 cells were attached to gold electrodes with different diameters to form a cell-NC membrane electrode system for impedance testing.

An equivalent circuit model of the GE, NGE and CNGE groups was established based on the acquired EIS data. We then used the equivalent circuit to fit the data, analyzed the circuit components, and extracted the key parameters (R_s_, CPE-T, CPE-P, and R_p_) by PCA as the input characteristics for SVR; the diameter served as the output for data training and prediction.

## Results

### Equivalent circuit analysis

From an electrical point of view, the performance of an electrochemical cell can be represented by an equivalent circuit that has the same behavior and output with an equivalent input [22–24]. To study the equivalent model of the biomembrane impedance sensor, the equivalent model of the impedance sensor without a biomembrane (GE) needed to be first analyzed [25, 26]. According to the principles of electrochemical theory, the electrochemical reaction of a solution comprises two processes: the diffusion of ions from the solution to the interface of the electrode, i.e., the mass transfer process, and the ion reacting on the electrode, i.e., the activation process [6, 27, 28].

**Fig. 3:**
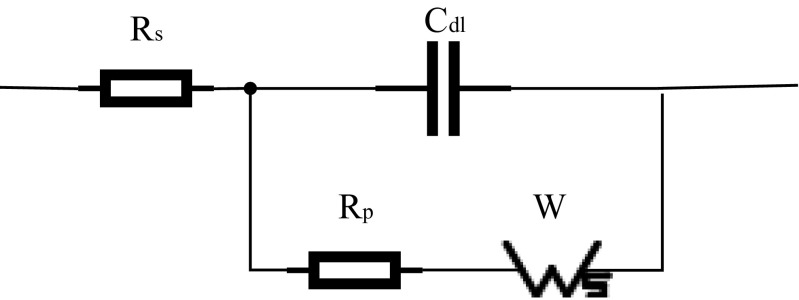
The Randles equivalent circuit model

To simplify a complex system, such as electrodes in contact with different electrolytes, the model of the impedance sensor can be considered equivalent to the classical Randles model [25, 29, 30] (Fig. 3). The circuit model comprises four elements: the ohmic resistance of the electrolyte solution (R_s_); the capacitance at the solution interface near the electrode, also known as the double-layer capacitance (C_dl_); the Warburg impedance (W); and the charge transfer resistance (R_p_).

**Fig. 4:**
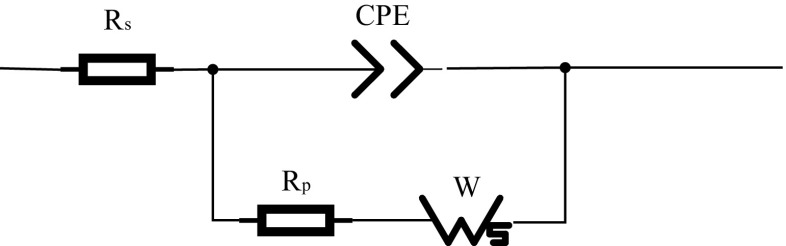
Practical equivalent circuit model of the GE

The Faraday impedance of the equivalent electrolyzer circuit for charge-transfer control and diffusion control includes two parts: W and the electrode polarization resistance, R_p_. Because the gold electrode used in this paper is an inert electrode, the Faraday impedance is very large. Thus, in this circuit, R_s_ and C_dl_ are approximated as ideal circuit elements, and the R_p_ and W impedances are not ideal components; they have a certain relationship with the measuring frequency. Additionally, because the electrode surface was rough, we replaced the ideal capacitor element (C_dl_) with a constant phase element (CPE) [26, 29], which can improve the quality of the fitting (Fig. 4).

Compared with the GE, the NGE has an NC membrane that participates in ion exchange; thus, the equivalent circuits of the GE and NGE differ. The working electrode in this experiment was separated from the other two electrodes with an NC film, which is equivalent to a shielding layer on the working electrode; thus, the oxidized and reduced substances were prevented from exchanging electrons on the electrode [28, 30]. Normally, R_p_ depends on the dielectric and insulating features at the electrode–electrolyte interface, but under these conditions, it was affected by the property changes occurring at the interface. Compared with GE, the immobilization of the nitrocellulose membrane changed the dielectric features at the interface, resulting in an increase in R_p_. However, the capacitance between the metal and the membrane could be regarded as a CPE; thus, it was still equivalent to the CPE [31, 32]. In the equivalent circuit model of the NGE, the primary elements did not change; therefore, the NGE model was still equivalent to the equivalent model in Fig. 4.

The structure of the CNGE is similar to that of NGE; the difference between the CNGE and NGE is that MCF-7 cells are growing on the NC membrane in the CNGE. The NC membrane with cells can be regarded as a biofilm structure due to the adherence of cells. Thus, the equivalent circuit model of the CNGE can also be equivalent to the model in Fig. 4, but the resistance and the capacitance have changed. When the system applied a voltage to the cell-NC +membrane electrode, the current flowed through the electrode-NC membrane-cell system to reach the electrolyte. Thus, the resistance of the electrode-NC membrane cell could be considered to be in series with the resistance of the electrolyte, which can be described as resistance (R_p_) [28]. The capacitance between the metal and the cell-NC membrane can also be regarded as a constant (CPE) [30, 32]. According to the electrical characteristics, R_p_ will increase, and the change in the constant will be more rapid.

**Fig. 5:**
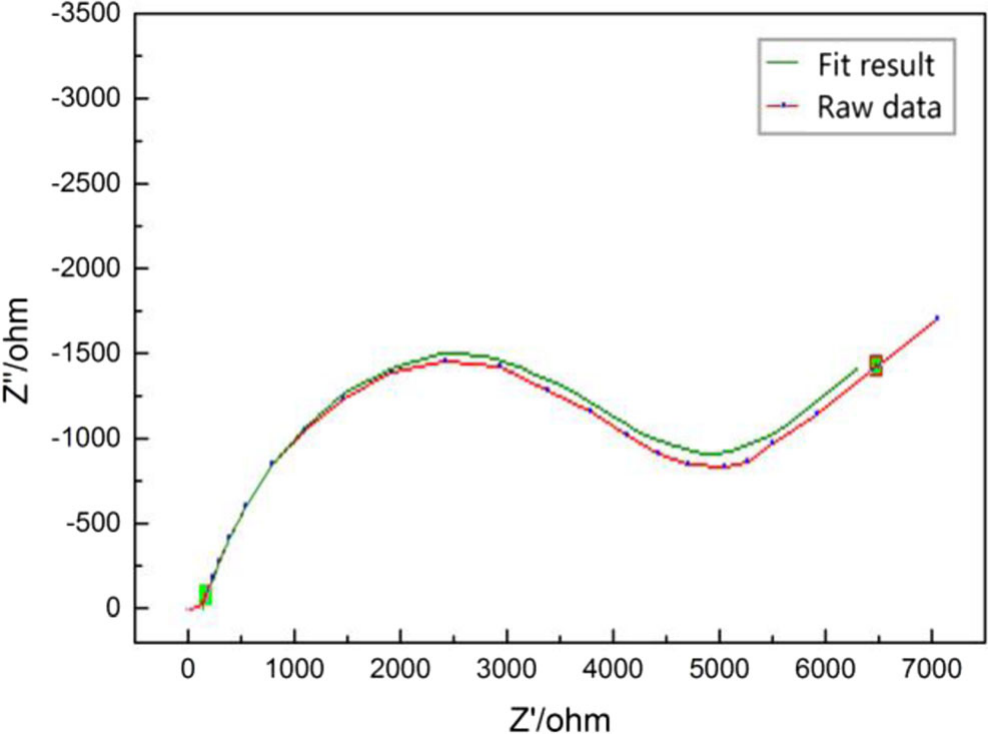
Comparison between the raw data and the fitting data

To obtain the values of the parameters in the equivalent model, the impedance data were fitted with the equivalent model in ZView (Fig. 5), as previously discussed. The comparison between the raw data and the fitting data demonstrates the practical application of this method.

As shown in Fig. 4, the equivalent model we used was composed of 4 elements, and 7 electrical parameters (R_s_, CPE-T, CPE-P, R_p_, W-T, W-P, and W-R) were generated. If the 7 parameters were all taken as inputs, the EIS analysis would be a very complicated 7-dimensional problem. To simplify this complex problem, we first used PCA to address the 7 parameters, and then, we only analyzed the 4 parameters most related to the output. In this way, the 7-dimensional problem was simplified to a 4-dimensional problem, greatly reducing the workload while only slightly affecting the accuracy. The specific PCA is discussed in detail in Section 3.2.

### Analysis of the characteristic parameters

**Fig. 6:**
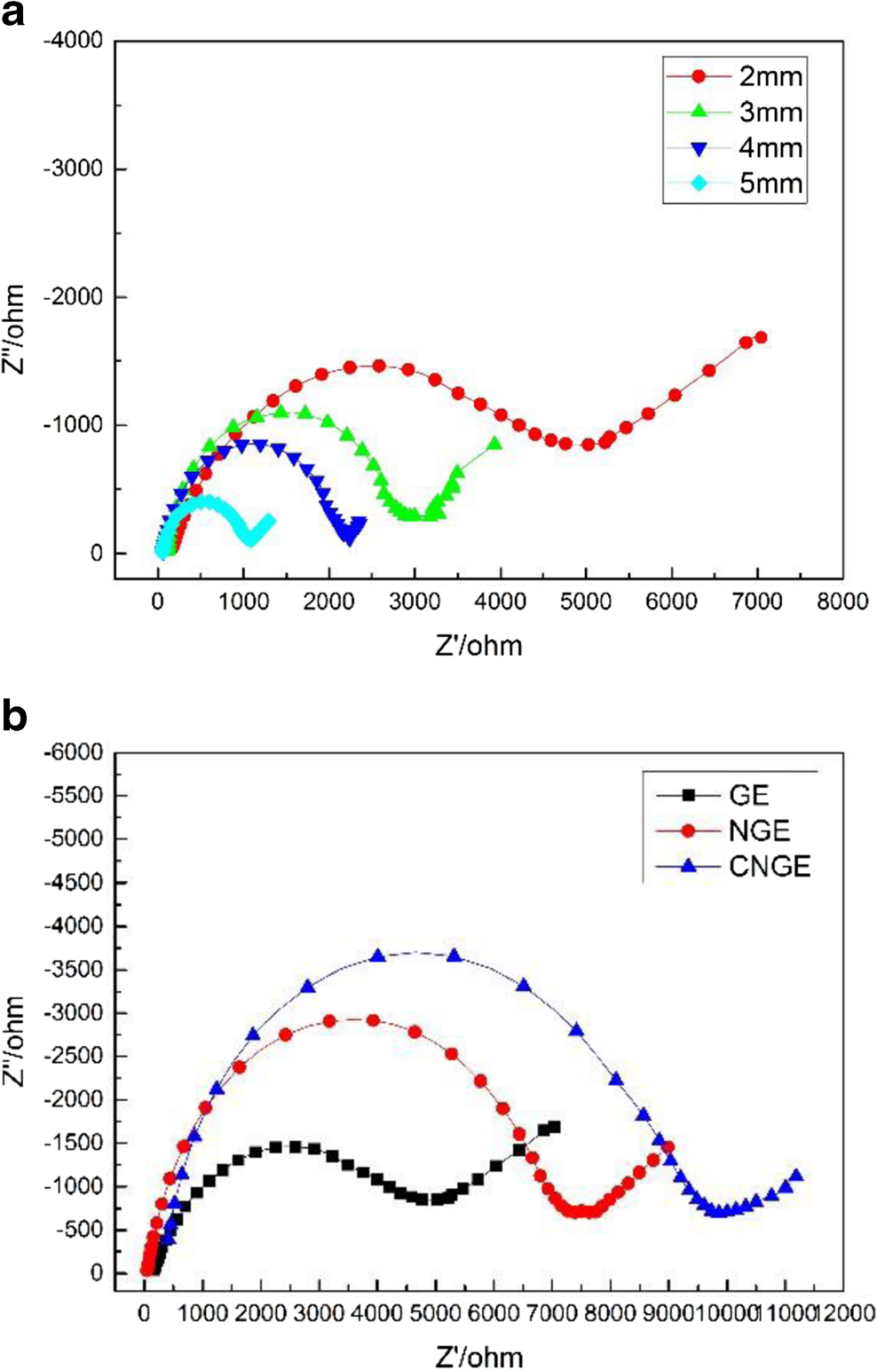
(**a**) The impedance spectra of GEs with different diameters (2 mm, 3 mm, 4 mm, and 5 mm) (**b**) The impedance spectra (Nyquist plots) of the GE, NGE, and CNGE with a gold electrode (2 mm)

The impedance spectra (Nyquist plots) of GEs with different diameters (1 mm, 2 mm, 3 mm, 4 mm, and 5 mm) are shown in Fig. 6(a). With increasing electrode diameter, the impedance semicircle decreases in magnitude and diameter. The impedance spectra (Nyquist plots) of the GE, NGE, and CNGE are shown in Fig. 6(b). The impedance spectra are characterized by a semicircle portion at high frequencies, corresponding to the electron transfer-limiting process [6, 29]; the diameter of the semicircle is equal to the charge transfer resistance. When different substances are adsorbed onto an electrode surface, they disturb the charge-transfer process between the electrode and the electrolyte solution, resulting in changes in the charge transfer resistance. As shown in the figure, the diameter of the semicircle increased when the nitrocellulose membrane and MCF-7 cells were immobilized on the electrodes.

After fitting the Randles model, we obtained 7 parameters (R_s_, CPE-T, CPE-P, R_p_, W-T, W-P, and W-R) [29, 30]. If all 7 parameters were used for regression prediction by SVR, the amount of data would be too large, and the operation speed would be too slow; such a large number of parameters would result in redundancy and a cumbersome analysis of the parameters. Therefore, PCA was used to analyze the correlation among the 7 parameters [31, 32], and the most relevant parameters were considered the main components. The information for the 7 parameters, obtained by the princomp function in MATLAB, is shown in Table 1.

**Table 1:** Parameter information for the electrochemistry experiments

Parameter	R_s_	CPE-T	CPE-P	R_p_	W-T	W-P	W-R
Proportion (%)	48.75	19.03	13.71	10.50	5.52	2.01	0.39

The four parameters with the highest relevance values of 48.75%, 19.03%, 13.71%, and 10.50% were R_s_, CPE-T, CPE-, and R_p_, respectively. These results are consistent with the aforementioned equivalent circuit and verify the practical significance of the equivalent circuit.

The amount of information contained in the four parameters was 91.99%, covering more than 80% of the circuit information. Thus, R_s_, CPE-T, CPE-P, and R_p_ can be selected as eigenvectors and used as input for SVR training and prediction.

### Analysis of data training and prediction

**Fig. 7:**
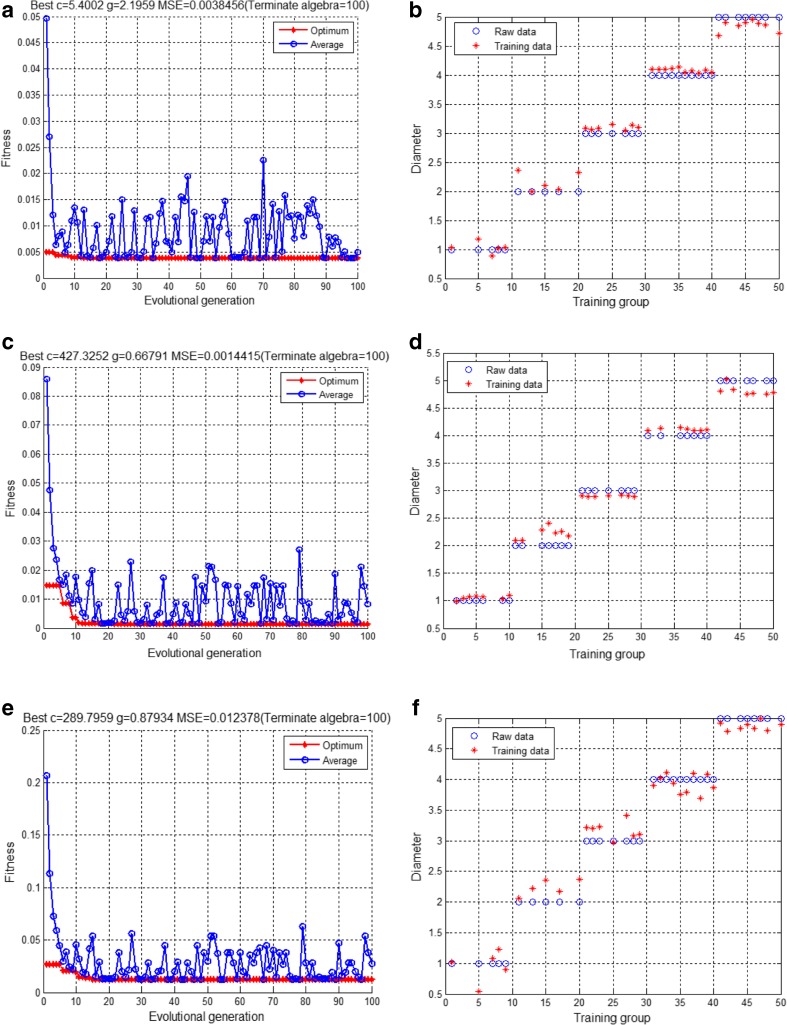
(**a**) Training of the GE, (**b**) training results for the GE, (**c**) training of the NGE, (**d**) training results for the NGE, (**e**) training of the CNGE, and (**f**) training results for the CNGE

**Fig. 8:**
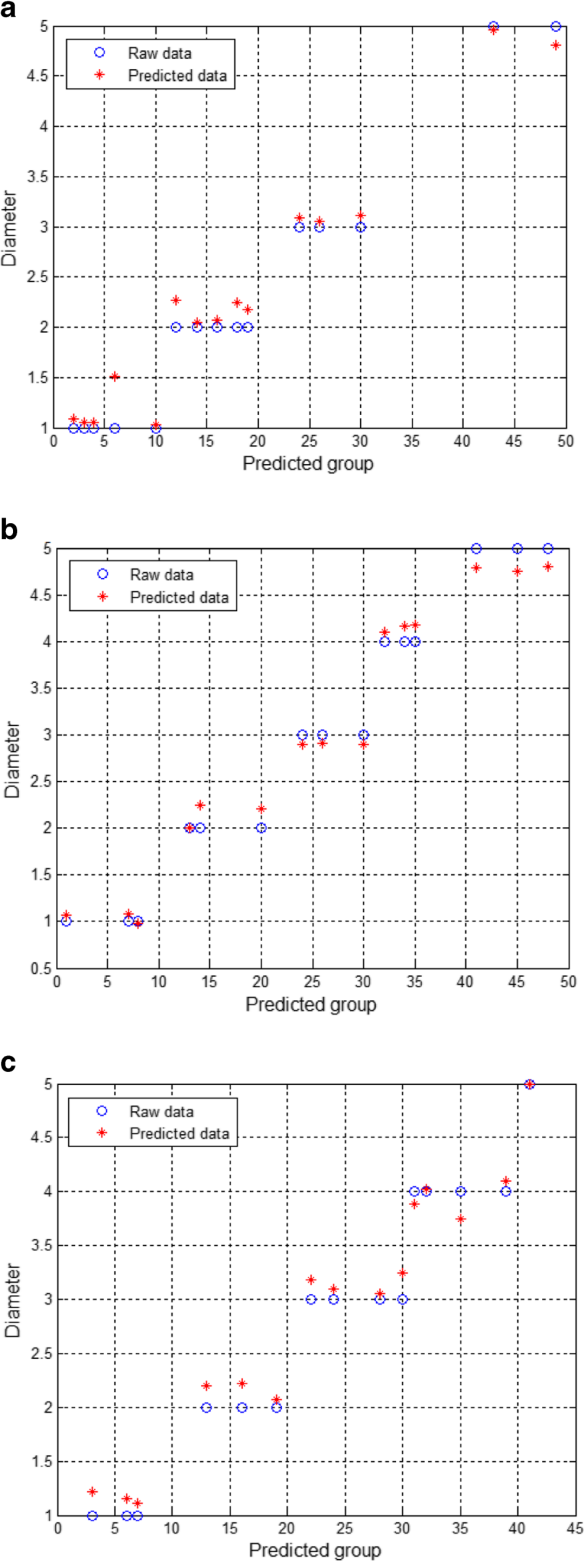
(**a**) Prediction for the GE, (**b**) prediction for the NGE, (**c**) prediction for the CNGE

R_s_, CPE-T, CPE-P, and R_p_ were used as inputs in the SVR model, and the electrode diameter was used as the output to establish the SVR model of the electrode. For each of the three groups (GE, NGE, and CNGE), 100 data points were collected, corresponding to 20 data points for each diameter (1 mm, 2 mm, 3 mm, 4 mm, and 5 mm) in each group. A total of 35% of the data points were randomly selected as the training data (Fig. 7), with 15% used as the prediction data (Fig. 8). The results obtained from each test were compared with the actual results to determine the mean squared error (MSE), training error rate, and prediction error rate (Table 2). To ensure the best results in the training process, the best result was automatically adjusted by the program. Moreover, random extraction, training, and prediction of the training data ensured that the differences among the data obtained for each group had little effect on the overall results.

**Table 2:** Training results and analysis from 10 tests of the three electrical impedance models

Model	MSE	Training error %	Prediction error %
GE	3.68E−04	5.19	3.70
	3.08E−04	4.43	7.71
	4.03E−03	5.24	4.06
	3.10E−03	5.42	3.01
	3.06E−03	5.06	5.76
	4.81E−03	5.35	3.61
	5.45E−04	4.74	4.81
	2.54E−03	4.09	7.29
	4.70E−04	5.09	3.72
	6.37E−04	4.97	5.34
Average	1.99E−03	4.96	4.90
NGE	7.92E−04	8.60	12.27
	9.26E−04	6.36	9.44
	1.05E−03	8.68	11.57
	5.34E−03	7.55	7.67
	4.07E−03	7.94	14.60
	3.44E−03	7.48	16.81
	9.32E−04	8.89	12.64
	2.91E−03	8.89	8.02
	7.11E−04	8.57	7.82
	7.92E−04	8.60	12.27
Average	2.24E−03	8.11	11.20
CNGE	6.12E−03	10.25	13.55
	1.18E−02	13.66	7.82
	2.01E−02	13.01	13.39
	1.76E−02	12.94	14.01
	2.16E−02	12.61	13.99
	9.48E−03	12.53	16.44
	7.58E−03	10.78	14.13
	1.37E−02	13.17	12.01
	7.57E−03	11.17	16.88
	1.35E−02	11.80	10.85
Average	1.29E−02	12.19	13.31

To randomly select 10 sets of data from each group, the three groups of impendence models were tested 50 times. On the basis of these selected data, 10 sets of data were tabulated. The three data sets contained the results for SVR implementation of impedance parameters for the GE, NGE, and CNGE. For the GE, the average MSE was 1.99E−03, the average training error was 4.96%, and the average prediction error was 4.90%. For the NGE, the average MSE was 2.24E−03, the average training error was 8.11%, and the average prediction error was 11.20%. For the CNGE, the average MSE was 1.29E−02, the average training error was 12.19%, and the average prediction error was 13.31%. As shown in the table, the average MSE of the GE was 1.99E−03, and its average training error and average prediction error were both less than 5%; thus, we inferred that its accuracy reached 95%. The average MSE values of the other two groups were approximately 2.24E−03 and 1.29E−02, and both the average training error and average prediction error were approximately 10%, which demonstrate that these four electrochemical parameters are reasonable and effective for setting up SVR models. We also concluded that the fitted model has good stability and prediction performance under different experimental conditions. As such, it can predict the repetition rate difference of the effective area on the electrode array or alternatively provide a method for predicting the active surface area of electrodes with different shapes but the same area or the effective cell-attached active surface area in cell attachment experiments with different densities.

## Discussion

In this paper, SVR was adopted to train and predict an evaluation model of a biomembrane electrode. The impedances with five effective electrode areas were tested under three different conditions (GE, NGE, and CNGE), and the electrochemical impedance spectra were fitted by modeling the corresponding equivalent circuits to determine the correlation between the effective electrode area and various electrochemical parameters. Based on PCA, key parameters (R_s_, CPE-T, CPE-P, and R_p_) were extracted as the input characteristics for SVR, and the effective electrode area was used as the output to train and predict the data. The average error of the three groups was 9.8%, that of the GE group was 4.9%, and the average MSE was 5.71E−3, which shows that the developed SVR model is accurate and widely applicable. We also expect that this model can be used to address both the problem of cell growth density on high-flux electrode arrays and the issue of determining the repetition rate of cell attachment on electrode arrays through a simple impedance test and SVR model. These problems are usually addressed through microscopic observations. This model can also aid in the quantitative analysis and evaluation of the exact cell attachment rate of electrodes with different shapes but the same area (such as interdigital electrodes, disc electrodes, and diamond electrode arrays).

## Conclusion

In conclusion, in this work, an electrochemical impedance biosensor based on SVR was developed to detect the effective area of a biomembrane. Under three different conditions (GE, NGE, and CNGE), the average prediction error of our biosensor was 4.9%. This result indicates that the biosensor in this paper can provide a sensing platform to selectively and quantitatively detect the effective areas of different types of biomembranes. These results might help further work on the rapid detection of cell growth status and could even be used for detection and analysis in multidirectional microorganism-related fields.

## Glossary

CNGE: Gold electrode with a cell-nitrocellulose membrane
EIS: Electrochemical impedance spectroscopy
GE: Bare gold electrode
NC-membrane: Nitrocellulose membrane
NGE: Gold electrode with a nitrocellulose membrane
PCA: Principal component analysis is a statistical procedure that uses an orthogonal transformation to convert a set of observations of possibly correlated variables into a set of values of linearly uncorrelated variables called principal components.
SVM: A support vector machine (SVM) is a machine learning algorithm with a wide range of applications; it is especially good at building and forecasting binary classification models. Support vector regression (SVR) is a regression method based on an SVM.
SVR: Support vector regression
